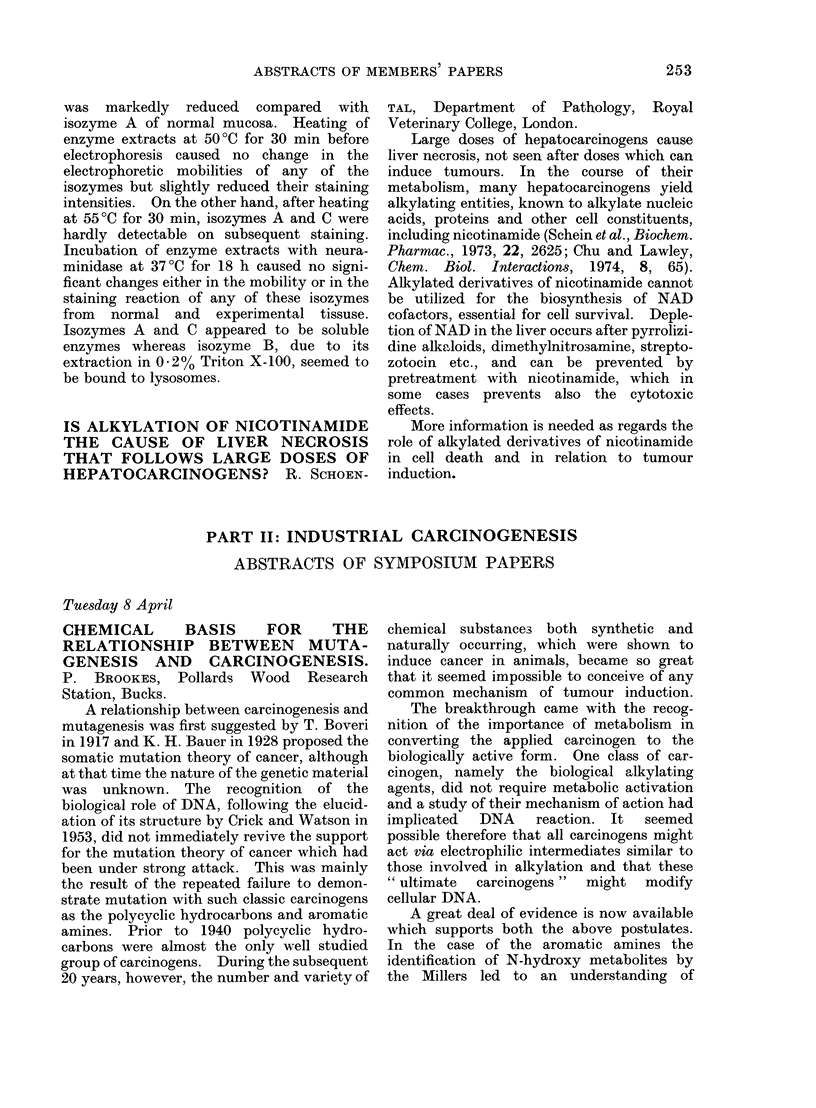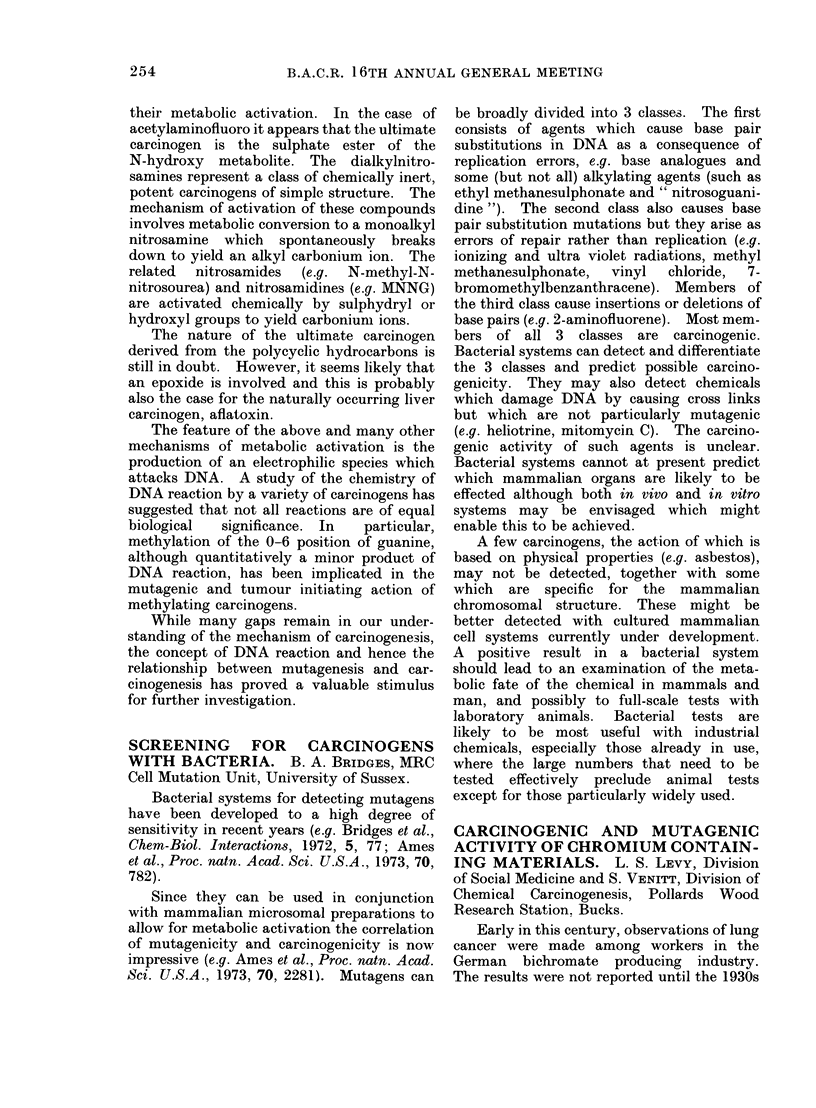# Proceedings: Chemical basis for the relationship between mutagenesis and carcinogenesis.

**DOI:** 10.1038/bjc.1975.199

**Published:** 1975-08

**Authors:** P. Brookes


### PART II: INDUSTRIAL CARCINOGENESIS

### ABSTRACTS OF SYMPOSIUM PAPERS

*Tuesday 8 April*

**CHEMICAL BASIS FOR THE RELATIONSHIP BETWEEN MUTAGENESIS AND CARCINOGENESIS.** P. Brookes, Pollards Wood Research Station, Bucks.

A relationship between carcinogenesis and mutagenesis was first suggested by T. Boveri in 1917 and K. H. Bauer in 1928 proposed the somatic mutation theory of cancer, although at that time the nature of the genetic material was unknown. The recognition of the biological role of DNA, following the elucidation of its structure by Crick and Watson in 1953, did not immediately revive the support for the mutation theory of cancer which had been under strong attack. This was mainly the result of the repeated failure to demonstrate mutation with such classic carcinogens as the polycyclic hydrocarbons and aromatic amines. Prior to 1940 polycyclic hydrocarbons were almost the only well studied group of carcinogens. During the subsequent 20 years, however, the number and variety of chemical substances both synthetic and naturally occurring, which were shown to induce cancer in animals, became so great that it seemed impossible to conceive of any common mechanism of tumour induction.

The breakthrough came with the recognition of the importance of metabolism in converting the applied carcinogen to the biologically active form. One class of carcinogen, namely the biological alkylating agents, did not require metabolic activation and a study of their mechanism of action had implicated DNA reaction. It seemed possible therefore that all carcinogens might act *via* electrophilic intermediates similar to those involved in alkylation and that these "ultimate carcinogens" might modify cellular DNA.

A great deal of evidence is now available which supports both the above postulates. In the case of the aromatic amines the identification of N-hydroxy metabolites by the Millers led to an understanding of their metabolic activation. In the case of acetylaminofluoro it appears that the ultimate carcinogen is the sulphate ester of the N-hydroxy metabolite. The dialkylnitrosamines represent a class of chemically inert, potent carcinogens of simple structure. The mechanism of activation of these compounds involves metabolic conversion to a monoalkyl nitrosamine which spontaneously breaks down to yield an alkyl carbonium ion. The related nitrosamides (*e.g.* N-methyl-N-nitrosourea) and nitrosamidines (*e.g.* MNNG) are activated chemically by sulphydryl or hydroxyl groups to yield carbonium ions.

The nature of the ultimate carcinogen derived from the polycyclic hydrocarbons is still in doubt. However, it seems likely that an epoxide is involved and this is probably also the case for the naturally occurring liver carcinogen, aflatoxin.

The feature of the above and many other mechanisms of metabolic activation is the production of an electrophilic species which attacks DNA. A study of the chemistry of DNA reaction by a variety of carcinogens has suggested that not all reactions are of equal biological significance. In particular, methylation of the 0–6 position of guanine, although quantitatively a minor product of DNA reaction, has been implicated in the mutagenic and tumour initiating action of methylating carcinogens.

While many gaps remain in our understanding of the mechanism of carcinogenesis, the concept of DNA reaction and hence the relationship between mutagenesis and carcinogenesis has proved a valuable stimulus for further investigation.